# Supplementary material for: Combining natural language processing and metabarcoding to reveal pathogen-environment associations
Source: PLoS Negl Trop Dis. 2021 Apr 7;15(4):e0008755. doi: 10.1371/journal.pntd.0008755 (PMC8055023; doi:10.1371/journal.pntd.0008755)
Supplement: Attachment — Submitted filename: revision_letter-1.docx [file pntd.0008755.s001.docx]

David Molik

250 Hesburgh Libraries

Notre Dame. IN 46556

(716) 982-5235
dmolik@nd.edu

Jan 18, 2021

Re: Resubmission of manuscript *Combining Natural Language Processing and Metabarcoding to Reveal Pathogen-Environment Associations*

The Editors

PLOS Neglected Tropical Diseases

1160 Battery Street, Suite 225

San Francisco, CA 94111

Dear Editors: Thank you for the opportunity to revise our manuscript, *Combining Natural Language Processing and Metabarcoding to Reveal Pathogen-Environment Associations*. We appreciate the careful review and constructive suggestions. It is our belief that the manuscript is substantially improved after making the suggested edits.

The authors believe that realigning the emphasis, as is alluded to by Reviewer One and whose original intention was understood by Reviewer Three, greatly improves the narrative of the paper. The results of the work are largely confirmatory, but the way that the authors reach those conclusions are novel and unique.

Several of the comments that the reviewers shared referred to our use of improper grammar, and our odd choices in language, these have been clarified for the readers’ benefit. A few comments alluded to a lack of geographical context. The authors added a new figure and geolocated and mapped C neoformans from the literature studied in our analysis, which adds geographical context.

Following this letter are the reviewer comments with our responses, including how and where the text was modified. Changes made in the manuscript are marked using track changes. The revision has been developed in consultation with all coauthors, and each author has given approval to the final form of this revision.

Thank you for your reconsideration.

Sincerely,

David C Molik

Enc.

**Reviewer's Responses to Questions**

**Key Review Criteria Required for Acceptance?**

**As you describe the new analyses required for acceptance, please consider the following**

**Methods**

**-Are the objectives of the study clearly articulated with a clear testable hypothesis stated?**

**-Is the study design appropriate to address the stated objectives?**

**-Is the population clearly described and appropriate for the hypothesis being tested?**

**-Is the sample size sufficient to ensure adequate power to address the hypothesis being tested?**

**-Were correct statistical analysis used to support conclusions?**

**-Are there concerns about ethical or regulatory requirements being met?**

Reviewer #1: Methods applied in this study are appropriate but the limited number of sample analyzed could drive to wrong conclusions.

*The authors agree and have revised our summary to better address how the methods were chosen to align with pathogens understudied in the field and/or under-reported by name in the literature where a small sample of papers for analysis means careful strides must be taken to expand the sources available to literature searches through use of NCBI SRA to make an alternative approach and we have revised our conclusion to focus more on efficacy and limitations of this approach. While this sort of study could have used a neural network the results would have been almost certainly over fitted due to the data sparse amount of articles. LDA is a standard in NLP and would be recommended in this context.*

The first paragraph concerning methods is too long and sometimes difficult to understand.

I suggest to move the background about validation of the computer methodologies in the introduction motivating the choice of them.

*We acknowledge the confusion, while Figure 1 aids the reader by explaining the steps taken in the methods from sequence to literature search. As a result, we have moved the figure up in the body of the text.*

As introduction of methods I suggest to list briefly the different steps of the analysis as reported in Fig. 1 which will be then deeply described separately in the following paragraphs. Line 214. Change "were positive for C. neoformans" to "were specific for C. neoformans".

*We revised this grammatic error, see lines 175, 227*

Line 214. Add accession number of the sequences used as queries.

*SRAs and accession numbers of queries are in supplement. Updated line 227.*

Figure 2. Symbols in the legend have been converted in squares so it is not possible to follow the explanation of the diagram.

*We are working with PLoS to figure out the problem (not displayed as submitted), possibly an old version of Windows in the workflow swaps the symbol?.*

Line 290. Abbreviation PCA should be explained at the first citation.

*Fixed, now line 313*

Textbox 1, 2 and 3 should be removed since they add a lot of confusing information.

*We understand that the inclusion of text boxes is not standard across all PLoS papers. However, this is an option in this journal and we feel that this feature is very useful for this manuscript. The methodology of NLP, and the complexity of the analysis we used introduce a number of terms and concepts that are not necessarily familiar to the broad audience we want to reach. The text box allows us to include some of this detail for those readers who may be less familiar with this area and would like a primer. Readers more comfortable with the subject can easily by pass these boxes with no loss of content.*

I suggest just to report in the methods the concept and calculation of precision, recall, accuracy, F1, false positive and negative, and true positive and positive.

*While we agree that the statistics relevant to this comment are hard to read, narratively, and with the sort of presentation were going for, a case could be made for either.*

Reviewer #2: There is a significant amount of detail about various aspects of the method and lengthy explanations provided in text boxes. The methods seem appropriate, but the question being asked is somewhat vague and the application of the various methods is not always clear. It was not clear how the txt analysis was culled from 75,000 words down to the 25 presented in Figure 4. The authors chose Gensim python program to conduct the LDA topic model generation, but the authors do not state why this program was chosen; if there are alternatives or if it is considered the standard method for this type of analysis.

*There are a lot of filters, the key thing is that there a set of words that can distinguish between papers. If the number of words are similar from paper to paper and do not have statistical power to distinguish between papers, than they are thrown out. Non-frequently occurring words confuses the LDA model generation, so infrequently found words are erased from the text corpus.*

*Gensim was chosen as the scientific package of choice for its wide support and ease of use for a variety of machine learning application and use cases, in addition to its memory efficient architecture for multiple trials. (*[*https://pypi.org/project/gensim/*](about:blank)*)*

Reviewer #3: No new analysis required.

--------------------

Results

-Does the analysis presented match the analysis plan?

-Are the results clearly and completely presented?

-Are the figures (Tables, Images) of sufficient quality for clarity?

Reviewer #1: Figure 4. Have different colors of bars any meaning?

*The colors do not have specific meaning, figure description has been updated. (see line 422)*

Line 387. Gensim software should be first cited in the methods with a reference.

Lines 417-424.

*See line 279 and citation #35, it’s a confusing citation, but the one the authors of Gensim recommend.*

Topic 1 is not described.

*Topic one is the outgroup and so we felt that the topic did not need to be described in detail, in the LDA we interpreted it as a catch all group for the negative papers and so expected it not to carry significance.*

Random Forest Evaluation. In this paragraph results should be reported in detail.

*Results moved to Results (see updated lines 530-532, as well as 470-484)*

Figure 7. Which is the unit of the bar on the right-side of the picture?

*The unit is the number of papers, figure is now figure 8, updated to number of papers*

Figure 8. I suppose class 0 refer o negative papers and class 1 positive papers but this should be better explained.

*see updated description of figure 9 which clears up this confusion.*

Table 2. From your definitions precision is the proportion of true positives out of all the others. Therefore in this case 20/31= 0.64. Why you reported 0.71? If my calculation is right then also F1 score should be 0.77 (0.64+0.9 /2).

*This is a multi-label micro-roc calculation which causes this result.*

Reviewer #2: It is not clear what topics were used to model, as the authors list 25 potential topic words excluding stop words. They state that the actual model apparently had three topics, but it wasn’t clear which three were included. Or if they modeled each set using an overlapping 3 topic word model. The significance of Figure 6 is not clearly explained. The final model presented in Figure 7 identified 20 true positives out of 31 tested samples, which is only 65%. It is reported in Figure 8 that the micro-average of the two ROC curves is 0.72, but each of those have an area of 0.55, so it is not at all clear how they obtained an average of 0.72. They also do not explain the significance of either micro- or macro-average. The data are presented with very little attempt to explain how they inform the prediction of where C. neoformans might be found or what it means in the context of the question being asked.

*There is some confusion between the words that make up a topic, and what was used to model. In order to use the LDA algorithm to create the models we create a matrix of word counts per-paper. These word counts, only the 25 differentiating words, in their proportions are fed to LDA to cluster the papers and attach how much of each topic each paper has. We take the best assigned topic to be the truth for the paper, and then pull the words that differentiated that cluster. These words need to be extrapolated by the researchers with subject matter intuition based on the text corpus given to the model and words present in the topics. (see updated lines 479-481) The optimal model was a model that had 3 topics.*

*We then use decisions trees on the word matrix to assign papers to topics using decision trees this is a verification step, to show that our model can return the same sets.*

Reviewer #3: Additional characterization of the positive hits to include geographic location and/or change over time in geographic location or other information about how the sequencing sample was collected.

*Added new figure 4, geographic locations. See lines 402 – 407. figure 4 illustrates the how understudied and under-reported C. Neoformans is in the field and the literature – that the figure illustrates the idiosyncratic nature and sparsity and convenience of geolocated C Neoformans field work and how methods like ours expand an understanding of understudied pathogens is relevant especially for opportunistic pathogens.*

--------------------

Conclusions

-Are the conclusions supported by the data presented?

-Are the limitations of analysis clearly described?

-Do the authors discuss how these data can be helpful to advance our understanding of the topic under study?

-Is public health relevance addressed?

Reviewer #1: Lines 449-451. C. neoformans is worldwide distributed, it is not concentrated in Sub-Saharian area!!!!

*Added word “even” on line 523, the reviewer will also appreciate the new figure 4.*

Reviewer #2: It is difficult to ascertain what conclusions the authors have obtained from this study. They state that it provides support for the previously suggested linkage between C. neoformans and decomposing wood. This is neither a novel nor unexpected result. The link between C. neoformans and decomposing wood is not in doubt as there are dozens of papers that have identified C. neoformans in various environmental locations associated with rotting wood. The application of NLP is a somewhat novel approach, but the utility of this approach is not very apparent in the unconvincing predictive power of the model that they built. It seems that unless the metagenetic data collected includes information about sampling location, the approach in this paper is unlikely to provide any additional insight as to the environmental origins of the sequenced samples.

*Authors have updated the conclusions and methods. Author summary is updated emphasizes the aim of our work. See lines 50-52.*

Reviewer #3: Additional discussion about implications for public health would enhance the paper.

*See 605-609, addressing some implications, and an important open question about how knowledge about how the niche can play an important role in research.*

--------------------

**Editorial and Data Presentation Modifications?**

**Use this section for editorial suggestions as well as relatively minor modifications of existing data that would enhance clarity. If the only modifications needed are minor and/or editorial, you may wish to recommend “Minor Revision” or “Accept”.**

Reviewer #1: (No Response)

Reviewer #2: None

Reviewer #3: I'm not sure if the presence of text boxes and other insets fits with the style of the journal. The information given in the textboxes may need to be added into the main text of the article, or given in a glossary at the end.

*We believe that the way the PloS publishing platform handles text boxes will greatly reduce the confusion of where the work of the papers ends, and extra information begins. And that this added information will greatly benefit any reader not familiar with these topics touched on by the paper.*

--------------------

Summary and General Comments

Use this section to provide overall comments, discuss strengths/weaknesses of the study, novelty, significance, general execution and scholarship. You may also include additional comments for the author, including concerns about dual publication, research ethics, or publication ethics. If requesting major revision, please articulate the new experiments that are needed.

Reviewer #1: In general this study is interesting since it try to obtain new information throughout new computer methodologies able to analyze a big amount of complex data.

On the other hand it presents a lot of steps which present a multitude of variables difficult to control and that can compromise the final result of the analysis.

*The authors appreciate the comment. This type of work requires careful attention to variables that can be difficult to control. Several methods not relevant to the article were tried and many were unsuited to this analysis. This paper’s drives have been made more apparent with the help of the reviewers’ comments. This method is novel, but as more articles like this come out, the methodologies will be tuned and automated.*

Reviewer #2: This manuscript presents a potentially novel method for identifying the environmental source of metagenetic data in the short-read archive at NCBI. The proposed method uses natural language processing to evaluate the associated manuscripts for information regarding where samples are were taken. The hypothesis is that sampled data may include genetic data from organisms in addition to those that were intended to be samples. In this study, the authors identify C. neoformans genetic material in SRA Biosamples that had not previously been shown to contain C. neoformans data. However, their results provide no additional information about the environmental source of these data other than that they were found in samples associated with decomposing wood, which is a well-established niche for C. neoformans. They do not make a convincing argument that this method will provide additional insight over what has probably already been collected and provided at the time of

sample collection.

*This comment was extremely helpful to the authors, having felt that the main drive of the paper, a novel method must have been missed a number of previously mentioned fixes have been implemented. Additionally, a new figure, figure 4 was added to further identify how these niches are novel.*

Reviewer #3: The manuscript “Combining Natural Language Processing and Metabarcoding to Reveal Pathogen-Environment Associations” by Molik et al is presents an interesting methodology which applies a Machine Learning approach to a specific scientific topic of interest, namely, the habitat of C. neoformans. While the paper is extremely well written and the concept is of interest to the field, I think there a few limitations that should be addressed, and if overcome would enhance the reception of the paper.

In the discussion, the author implies that the current consensus in the field is that C. neoformans is more abundant in Sub-Saharan Africa (Line 450) which is certainly not the case. It is well understood that while cases of cryptococcosis occur mainly in sub-Saharan Africa, this is due to HIV-AIDS spread and in these areas, not due to additional presence of the yeast. C. neoformans is known to be cosmopolitan, and has been found in the environment all over the world, which is discussed elsewhere in the paper.

*We believe the reviewer will be pleased to see Figure 4. And additional language throughout pointing to a global distribution.*

The overall conclusion of the paper, that C. neoformans is associated with soils and decomposing wood, seems to not add anything that was not previously known. The idea behind the paper and the methodology leading up to this conclusion is very exciting. If the positive hits could be further mined to add additional pieces of data such as where the positive hits were geographically located, and or the change in the geographic location over time it would strengthen the conclusion and add to what is already known. It would be interesting to know this data so that researchers can get more evidence for distribution and potential exposure and latency in individuals. While I think discussion of the technique and the implications for other pathogens is important, it is also important to provide more discussion about the topic selected for this paper. Some questions to consider in the discussion: How would understanding the ecological niche benefit the Cryptococcus field, what else could be revealed by future analyses with a larger amount of papers going forward, could this method also be applied to microbiome sequencing data to ascertain potential age/ area of exposure to C. neoformans? Overall I think the approach has a lot of promise and the authors have done a great job of explaining their method and taking a unique approach to investigating a pathogens origin.

*We find this comment inline with their own sentiments, and believe the reviewer will be pleased to note the changes on lines 50-52 reemphasizing why this work is important, in much the same way the reviewer feels.*
