# Supplementary material for: Combining natural language processing and metabarcoding to reveal pathogen-environment associations
Source: PLoS Negl Trop Dis. 2021 Apr 7;15(4):e0008755. doi: 10.1371/journal.pntd.0008755 (PMC8055023; doi:10.1371/journal.pntd.0008755)
Supplement: Attachment — Submitted filename: revision_letter_minor_revision.docx [file pntd.0008755.s002.docx]

David Molik

250 Hesburgh Libraries

Notre Dame. IN 46556

(716) 982-5235
dmolik@nd.edu

March 3, 2021

Re: Minor revisions of manuscript *Combining Natural Language Processing and Metabarcoding to Reveal Pathogen-Environment Associations*

The Editors

PLOS Neglected Tropical Diseases

1160 Battery Street, Suite 225

San Francisco, CA 94111

Dear Editors: Thank you for accepting with minor revisions our manuscript, *Combining Natural Language Processing and Metabarcoding to Reveal Pathogen-Environment Associations*. We have noted that reviewer one’s summary and general comments contains are the only comments which require addressing, as such we have enclosed only reviewer one’s summary and general comments.

Reviewer one makes two notes for revision, a line change due to an incorrect findings over a statement on Sub-Saharan Africa, we have implemented the reviewers suggestion, and a general comment about figure legends lacking.

Sincerely,

David C Molik

Enc.

**Reviewer One states:**

**The authors replied correctly to most of the comments but one point concerning conclusions still remains to be revised.**

**Sentence from line 567 to line 570 should be deeply modified as follows:
“Our results show that there is a link between C. neoformans and wood decomposition confirming that this fungus lives in the environment as saprophyte with a preference for wood substrates. This characteristic make C. neoformans able to occupy a multitude of environmental niches worldwide. These conclusions confirm the validity of the methodology here applied. Our de novo…..”

Statement about findings of C. neoformans outside Sub-Sahara is not correct and should be deleted.

Figure legends are lacking.**

In order to address these two comments, reviewer’s one suggestion for lines 567-570 was implemented, although this now corresponds to lines 486-489 in the new version of the manuscript. We feel that this is not only a better way to explain our ideas but also cleans up any confusion around comments on Sub-Saharan Africa.

In order to address the “Figure legends are lacking comment””

we added lines:

- 205-207 summarizing figure one
- 422-423 explaining how we use the Coherence and Perplexity results.
- 480-481 interpreting the results of the random forest model
